# Renuvion RF-Helium Plasma for Subdermal Skin Tightening, Facial Contouring and Skin Rejuvenation of the Face and Neck

**DOI:** 10.1089/fpsam.2020.0070

**Published:** 2020-07-02

**Authors:** Richard D. Gentile

**Affiliations:** ^1^ Gentile Facial Plastic and Aesthetic Laser Center, Youngstown, Ohio, USA.; ^2^ Division Facial Plastic Surgery, Cleveland Clinic Akron General Hospital, Akron, Ohio, USA.

## What Is Renuvion/J-Plasma/Cool Helium Plasma?

Energy has been applied in some form to tissue since the beginning of recorded history. The practice of applying heat to tissue with the use of cauters was used for thousands of years as an invaluable method of controlling hemorrhage. Continuous improvement of methods for utilizing the beneficial effects of heat on tissue eventually led to the development of the basic concepts of electrosurgery we know today. One of the newer entrants to the energy-based device market is Renuvion^®^ (Apyx Medical, Clearwater, Florida), which is powered by helium plasma. We have been using this advanced energy device in our practice for ∼3 years and find it to have certain favorable features.^1–3^ The unique Renuvion energy—helium plasma and proprietary radiofrequency (RF)—allows for precisely controlled delivery of heat to tissue, with minimal thermal spread and rapid heating with near-instantaneous cooling in part aided by the cooling effect of helium gas under the skin (in subdermal applications), which allows for shorter duration of activation and, therefore, less diffusion of heat to the skin. Studies show that during subdermal use of Renuvion, temperature at the surface of the skin does not rise as significantly as with some other subdermal technologies and because of this, external temperature monitoring may not be a necessity although it may be beneficial for treatment observation for uniformity of treatment as well as a safety monitor to observe for unusually high temperature fluctuations. It is thought that the coupling of technology with tissue, Helium produced subdermal environmental change and thermodynamic changes facilitating adjacent tissue cooling phenomenon contribute to its unique properties in limited dermal and cutaneous increase of temperature. We will review three applications for Renuvion that will be important in facial plastic and reconstructive surgery.

## Ablative Skin Rejuvenation

**Fig. 1.**
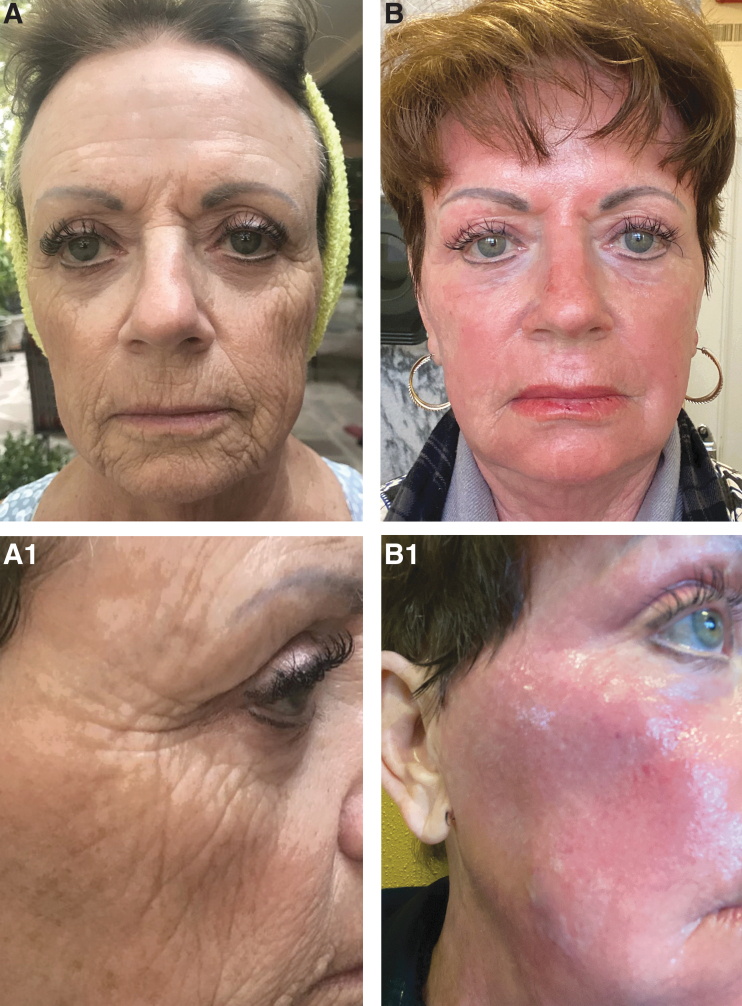
**(A)** This 75-year-old has severe actinic sun damage and is shown before her full face skin rejuvenation. **(B)** She is shown 1 month postprocedure with improvement in her cobblestone rhytids. She had treatment settings of 40% and 4 lpm helium reduced to 20% around the eyes. Two passes were made. **(A1)** Close up of lateral orbital region. **(B1)** One month postoperative Renuvion skin rejuvenation.

Ablative skin rejuvenation for deep rhytids and elastosis has in the past involved both laser and chemical alternatives. For years, phenol peeling and trichloroacetic acid were frequently used. The pendulum switched to lasers in about 1994 when we purchased our first laser. The pendulum then swung away from ablative treatments with newer technologies featuring fractionated or hybrid lasers. Renuvion represents the first device available for highly ablative skin rejuvenation. The FDA approval for Renuvion is for delivery of radiofrequency energy or helium plasma for cutting, coagulation, and ablation of soft tissue. A more specific indication for rhytid reduction is being pursued by the company to facilitate marketing for “wrinkle reduction.” The skin rejuvenation aspects of this technology were first noted by Dr. Joseph DeLozier a plastic surgeon in Nashville, Tennessee, who first introduced the freehand technique. With Renuvion treatment energies of 20–40% with helium flow rates of usually four are utilized for the treatment. Treatment can be done with either a painting, pulsed, or fractionated methods. The pulsed and fractionated treatment techniques were developed by Gentile and McCoy^3^ and represent a method of reducing energy treatment levels (morbidity) as this is a highly ablative treatment. One to two passes are typically done. An example of a patient undergoing Renuvion pulsed skin rejuvenation is shown in Figure 1. A short video of the pulsing technique is shown in Supplementary Video S1. Renuvion is also helpful for treatment of phymatous rhinophyma.^1,2^

## Subdermal Renuvion for Facial and Neck Contouring with Skin Tightening

**Fig. 2.**
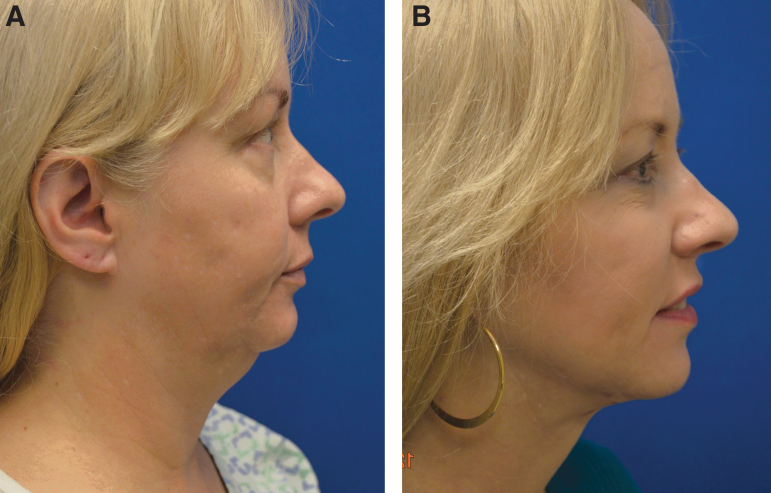
Fifty-eight-year-old concerned with submental fat and loss of mandibular contour. Preoperative view **(A)** and 4 months postoperative view **(B)** are shown with increased definition of jawline and jawline being evident. She also had medial platysmaplasty.

We introduced concepts and techniques for subdermal facial and neck rejuvenation in 2007 after being introduced to subdermal laser lipolysis concepts of skin tightening.^4–8^ at about the same time. These concepts were first considered controversial but have subsequently evolved with the advent of temperature-regulated monopolar, bipolar, and ultrasound devices that all work in the same paradigm thermal-mediated fibro liposculpture with remodeling. The evolution of devices has advanced from Smartlipo lasers to Precision TX to ThermiRF to FaceTite and now RF-helium plasma. Higher levels of temperature monitoring are implemented on the more recent devices, but as mentioned, only the Renuvion device has a self-limiting ability to avoid temperature extension to the dermis that can result in thermal burns. We utilize the subdermal facial and neck approach to reduce fat and tighten skin and soft tissue in the submental area mandibular angle and the jowls. Procedures can be done as a standalone (device only) technique or combined with minimally invasive surgical techniques (hybrid procedures). The settings for the device for subdermal procedures differ from the settings on the skin rejuvenation technique. Usually 75% power and 1.5 L of helium flow are used. The subdermal technique also involves more passes as typically six subcutaneous passes are done in the midline followed by left and right cervical and then left and right facial if indicated for jowl reduction. A video highlighting the facial and neck treatment is shown in Supplementary Video S2 and preoperative and postoperative photos of a patient undergoing a nonexcisional face and neck treatment are shown in Figure 2.

## Conclusions

Thermoplastic techniques for rhytidectomy and the holy grail of the “Shrink Wrap” facelift continue to evolve and improve with the advent of new technologies such as Renuvion RF-Helium Plasma. Although these techniques are not positioned to replace facelift surgery, they do offer a suitable alternative to those patients who refuse rhytidectomy surgery.

## Supplementary Material

Supplemental data

Supplemental data
